# Time-Series Assessment of Camp-Type Artisanal and Small-Scale Gold Mining Sectors with Large Influxes of Miners Using LANDSAT Imagery

**DOI:** 10.3390/ijerph18189441

**Published:** 2021-09-07

**Authors:** Satomi Kimijima, Masayuki Sakakibara, Masahiko Nagai, Nurfitri Abdul Gafur

**Affiliations:** 1 Research Institute for Humanity and Nature, Kyoto 603-8047, Japan; sakaki@chikyu.ac.jp; 2 Graduate School of Science & Engineering, Ehime University, Matsuyama 790-8577, Japan; 3 Graduate school of Science and Technology for Innovation, Yamaguchi University, Ube 755-8611, Japan; nagaim@yamaguchi-u.ac.jp; 4 Bappeda-Litbang Bone Bolango, Suwawa 96113, Indonesia; vivinurv3@hotmail.com

**Keywords:** artisanal and small-scale gold mining, gold price, Indonesia, influxes of miners, landcover change, mining camp, remote sensing

## Abstract

Mining sites development have had a significant impact on local socioeconomic conditions, the environment, and sustainability. However, the transformation of camp-type artisanal and small-scale gold mining (ASGM) sites with large influxes of miners from different regions has not been properly evaluated, owing to the closed nature of the ASGM sector. Here, we use remote sensing imagery and field investigations to assess ASGM sites with large influxes of miners living in mining camps in Bone Bolango Regency, Gorontalo Province, Indonesia, in 1995–2020. Built-up areas were identified as indicators of transformation of camp-type ASGM sites, using the Normalized Difference Vegetation Index, from the time series of images obtained using Google Earth Engine, then correlated with the prevalent gold market price. An 18.6-fold increase in built-up areas in mining camps was observed in 2020 compared with 1995, which correlated with increases in local gold prices. Field investigations showed that miner influx also increased after increases in gold prices. These findings extend our understanding of the rate and scale of development in the closed ASGM sector and the driving factors behind these changes. Our results provide significant insight into the potential rates and levels of socio-environmental pollution at local and community levels.

## 1. Introduction

The development of mining sites has had a significant impact on local socioeconomic conditions, the environment, and their sustainability. These developments have harmful socio-environmental consequences. Therefore, understanding the speed and scale of the development of mining sites and the factors driving these changes should provide significant insight into the potential rates and levels of socio-environmental pollution at local and community levels. This may also allow problems to be avoided and alternative sustainable strategies to be developed by involving various stakeholders at different levels.

The artisanal and small-scale gold mining (ASGM) sector is a significant gold-producing sector and is the largest employer in gold mining throughout the world. This sector represents ~20% (400–600 T/year) of the gold production and 90% of the gold-producing workforce on the global stage [1]. ‘ASGM’ refers to the mining practiced with rudimentary technology by an individual, group, or community [2]. The sector can be generally characterized as informal, unregistered, and illegal [3]. In this practice, gold is commonly extracted with mercury at the stage of amalgamation, causing extremely harmful environmental and occupational health hazards as a result of mercury pollution [4,5,6]. Mercury emissions into the atmosphere and its releases into water from ASGM are significant, and the ASGM was the dominant sector emitting mercury (37.7%) into the air in 2015 [5], mainly in South America, Africa, and Asia [4,7]. Other health problems, such as silicosis, methyl-orthophosphate-related poisoning, and various injuries, also occur during the mining process [8]. Despite the negative socio-environmental consequences, ASGM activities are still undertaken, predominantly in rural areas of >80 countries, as a significant poverty-alleviation mechanism, and to drive their economic development [1,3]. Indonesia, where the national poverty line is 9.4% as of 2019 [9], shows the continuous growth of ASGM across the country [10,11].

The Minamata Convention on Mercury, a global treaty protecting human health and the environment from anthropogenic emissions and the releases of mercury and its compounds [12], was adapted and came into force in October 2013 and August 2017, respectively [7]. Article 7 of the Convention focuses especially on ASGM sector, advocating the reduction of mercury use in the sector, and has been strategically taken up in national action plans and various national regulations among the ratifying nations [13]. However, the formalization of this policy has often been impeded by political issues, such as insufficient institutional frameworks, capacities, and funds [14,15]. In Indonesia, despite the formalization of the Convention and the development of a national action plan, the alternative supply of mercury has been domestically produced [16,17], resulting in a significant increase in informal mercury imports [18]. Therefore, it is expected that the generation of an alternative supply of mercury at a lower cost will accelerate ASGM and allow operators to expand their activities and the use of mercury beyond the regulated levels, as the price of gold on the global market increases [19]. A relationship between increases in ASGM and in gold price has been reported in the literature [20,21].

The ASGM sector can be categorized into two types: the ‘travel-type’, in which miners commute from their local residences to mining sites, and the ‘camp-type’, in which miners live and conduct their mining activities at mining camps. Recent research has focused mainly on the environmental and human health effects of mercury pollution caused by the travel-type ASGM sites spread over a large number of areas [2,8,22,23,24,25,26,27,28,29], largely employing the alluvial mining method. Although camp-type ASGM sites have also been studied [30], they have only been recorded as points on maps, and there has been no quantitative analysis of the changes in the camp-type mining sector over time.

As remote sensing technologies have developed, they have been widely used to characterize natural features and physical objects, allowing spatial changes in these to be monitored over time. Remote sensing also provides diverse continuous data with temporal, spatial, and spectral resolutions. Freely available satellite remote sensing data, such as Landsat series, have provided long-term datasets of Earth observations since the 1970s, and are extensively used to detect and monitor landcover [31,32,33,34]. The use of such long-term satellite datasets has allowed the development of qualitative and comprehensive understanding of various changes, including ASGM development [2,27,34,35]. Using remote sensing technologies, several studies have assessed ASGM-related qualitative spatiotemporal changes, such as deforestation, the extent of mining areas, and geomorphic and hydrological changes [21,27,28,29,35,36]. However, those studies were limited to ASGM areas with long and well-known mining histories, and mainly examined the travel-type part of the sector.

However, as the gold price has increased, artificially developed camp-type ASGM sites, spread across small areas, have developed in remote rural areas, with significant influxes of miners from neighboring regions [16,37,38]. The movement of such invisible informal influxes of miners to artificial camps causes informal communities to form and expand, accelerating the severe socio-environmental pollution inside the camps. However, this camp-type ASGM, with large influxes of miners, has never been quantitatively investigated in depth. Because camp-type ASGM in remote rural areas is associated with influxes of miners who live at the mining camps, the spatial distribution of built-up areas can be a significant indicator of the transformation of otherwise invisible ASGM communities.

In this study, our primary objective was to assess the transformation of the ASGM sectors by large influxes of miners living at mining camps in Bone Bolango Regency, Indonesia. Our specific objectives were: (1) to assess the landcover changes in 1995–2020, using remotely sensed imagery, such as a Landsat series; and (2) to correlate the ASGM-directed transformations with the prevalent gold price. The results of this study contribute to our understanding of the spread of camp-type ASGM activities spread across a small remote rural area and allow the rate and level of socio-environmental pollution in its train to be predicted.

## 2. Materials and Methods

### 2.1. Overall Methodological Workflow

**Figure 1:**
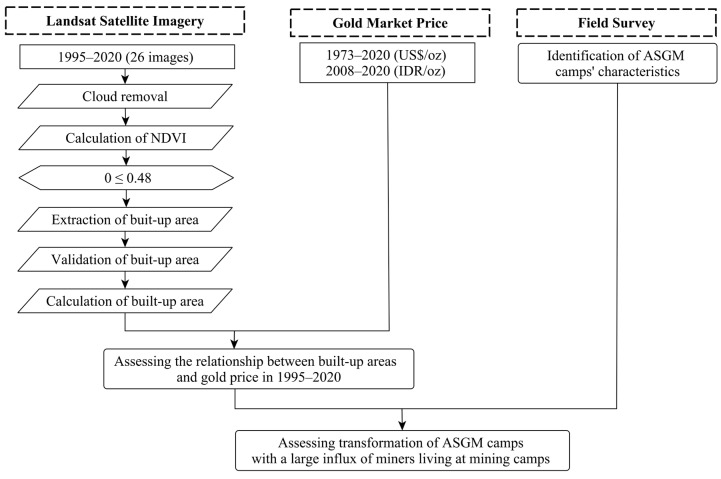
Overall methodology.

Figure 1 shows the methodological workflow used in this study. We focused on three significant steps to achieve our primary objective of assessing the transformation of ASGM with large influxes of miners into mining camps. First, the built-up areas in the mining camp in 1995–2020 were calculated from Landsat data. Second, the relationship between the identified built-up areas and the historical gold price was assessed. Third, a field survey was conducted to investigate the characteristics of the ASGM camps. Together, this evidence allowed us to understand the transformation of the ASGM sector by large influxes of miners living at mining camps. We present a discussion based on all the findings described. The methods used in each step are explained in the following sections.

### 2.2. Study Area

The North Sulawesi, Indonesia, is a well-mineralized metallogenic region, with significant gold mineralization associated with quartz veins, in a variety of porphyry and epithermal settings. The vertical tunnel method (shaft) of mining is predominantly used in the country, and the gold is extracted with mercury amalgamation in almost all of Indonesia’s ASGM hotspots [11], including in Sulawesi.

The Motomboto ASGM area is located ~30 km southeast of the city of Gorontalo in Bone Bolango Regency, Gorontalo Province, Indonesia. This area is categorized as having high-sulfidation epithermal deposits of copper, gold, and silver [39]. Gold mining in Bone Bolango Regency, Gorontalo began in the Dutch era (18th century) [40], and later, mining activity in the West Motomboto and Tulabolo areas was developed by Tropic Endeavour Indonesia in 1988 [41]. However, these mining sites were closed in 1991 because they intruded upon the Bogani Nani Wartabone National Park development [41]. The closure of the former mining site has triggered the entry of residents to the area to undertake mining activities [41]. In 2013, more than 9000 small-scale miners were reported in the Bogani Nani Wartabone National Park [38]. The majority of them came from neighboring regions, including Bolaang Mongondow and Minahasa in North Sulawesi, Indonesia [38].

**Figure 2:**
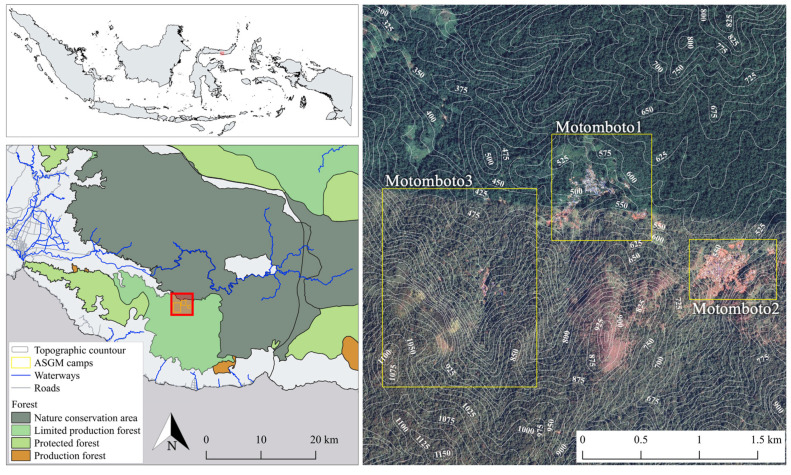
Study area.

In this study, we examined the Motomboto ASGM area, and divided it into mining camps 1, 2, and 3 (Figure 2). 

### 2.3. Satellite Imagery

Atmospherically corrected cloud-free Landsat data from the Thematic Mapper (TM), Enhanced Thematic Mapper Plus (ETM+) and Operational Land Imager (OLI), satellite images, available from the United States Geological Survey (USGS) through the Google Earth Engine, were used to extract a time series of built-up areas. The images were chosen based on cloud coverage and satellite data availability to minimize any potential influencing factors. Consequently, imagery from March–May was primarily selected. For those years in which data for the target months were not available, imagery collected in adjacent months was used. With these provisos, satellite imagery acquired from 1995 to 2020, with a ground resolution of 30 m in the World Geodetic System 84 (WGS84) geographic coordinate reference system, was used to detect and analyze the built-up areas in the study region.

In previous studies, the mining areas in Bone Bolango Regency were estimated to cover 0.62 km^2^ in 2012 [30]. Therefore, the long-term trend in ASGM sites could be detected with satellite imagery, even at a ground resolution of 30 m. The main specifications of the sensors used in this study are summarized in Table 1.

### 2.4. Extraction and Calculation of Built-Up Areas

Landsat satellite images acquired in 1995–2020 were used. Since the transformation by ASGM in remote rural areas is associated with influxes of miners who live at mining camps, as described in the Introduction, the spatial distribution of built-up areas was extracted as a significant indicator of the transformation of the ASGM camps resulting from large influxes of miners. Built-up areas can be defined by their physical aspects, such as predominantly human-constructed elements [42], as in this study. A number of spectral indices, including the Urban Index (UI) [43], Normalized Difference Built-up Index (NDBI) [44], Index-based Built-up Index [45], Built-up Area Extraction Method [46], Enhanced Built-up and Bareness Index [47], Band Ratio for Built-up Area [48], Built-up Index [49], Normalized Difference Vegetation Index (NDVI) [34], and Automated Built-up Extraction Index [50], have been developed to extract built-up areas from satellite imagery. Furthermore, human visual interpretation was also used [42]. Previous studies found that NDBI [51,52] and UI [53,54] are most sensitive in retrieving built-up areas, although these have mainly been used in urban studies. As NDBI and UI are incapable of efficiently separating built-up areas from bare land [44], the separation of these two land types in rural areas is more complicated. Therefore, in this study, we used NDVI, as used elsewhere [34], to analyze remote mining areas over long timescales. The value of NDVI in the built-up areas was calculated with Equation (1).

*NDVI* = (*NIR* − *Red*)/(*N*
*IR* + *Red*) (1)

NDVI, ranging from −1 to 1, shows a high value for dense vegetation and a low value for desert or unvegetated areas [55]. In this study, we further restricted the built-up areas using the NDVI threshold, 0 ≤ NDVI ≤ 0.48, to exclude vegetated areas on the land surface. The value was determined based on comparisons of the accuracy levels by referring high-resolution satellite data. In this way, the built-up areas were identified, and results were visualized as a time series. To assess the accuracy of the results, 100 points were randomly selected in the study area and validated using a high-resolution image obtained on 8 February 2017 using Google Earth Pro. Because no images were available in Google Earth Pro for the same dates as the Landsat imagery acquired from USGS, images acquired on the closest date (24 April 2017) were used. In this study, we applied the validated accuracy to all of the classification results, owing to the unavailability of reference data.

The relationship between the built-up areas and the gold price was also assessed. The global gold prices [56] and gold prices in Indonesian rupiah [57] were obtained for 1973–2020 and 2008–2020, respectively. They were then graphed against the total built-up area in the mining camps across time, and the correlation between the two parameters was calculated.

### 2.5. Investigation of ASGM Camps

Field observations of the Motomboto ASGM camps were made on 6 February 2020. Settlements, trommel machines, pools for immersing the materials, and miners’ camps were investigated. Interviews were also conducted with key miners in the mining camps.

## 3. Results

### 3.1. Expansion of Built-Up Areas in the Mining Camps

**Figure 3:**
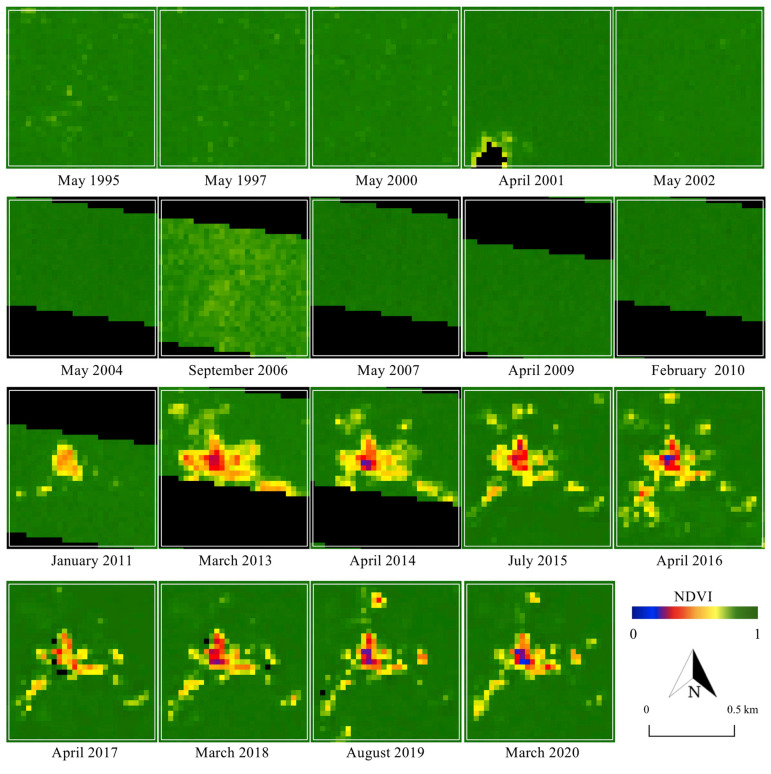
Changes in NDVI in Motomboto ASGM camp 1.

**Figure 4:**
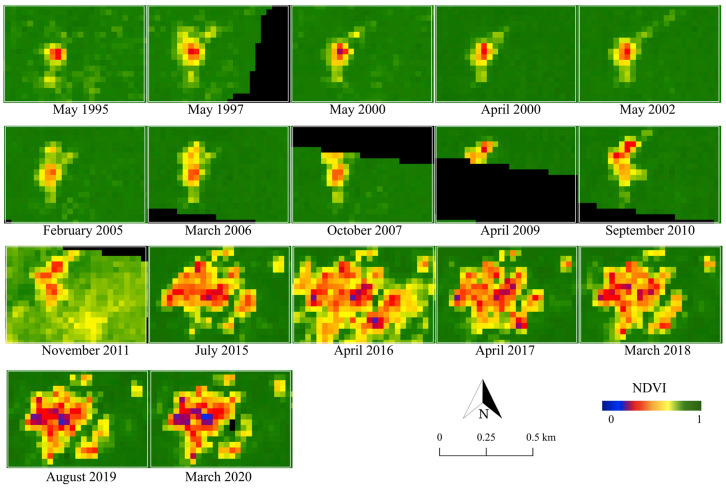
Changes in NDVI in Motomboto ASGM camp 2.

**Figure 5:**
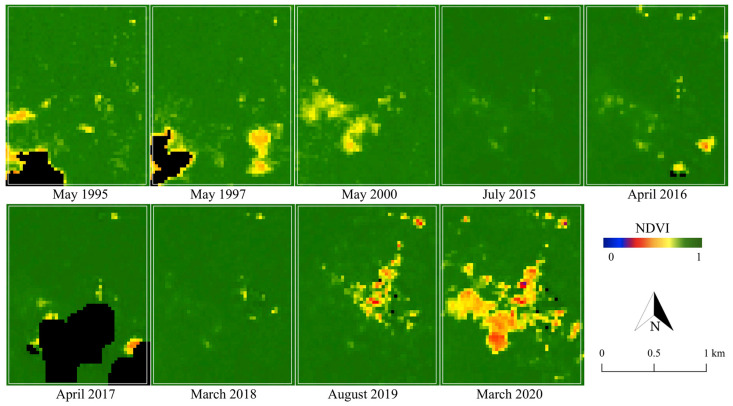
Changes in NDVI in Motomboto ASGM camp 3.

To detect the changes in the landcover surrounding the ASGM camps, NDVI was primarily calculated for 1995–2020. The appearance of changes and the rate of their development varied across the camps. Figure 3, Figure 4 and Figure 5 show the changes in NDVI in each camp over the 25-year study period. Motomboto ASGM camp 1 was first identified around 2011. However, it did not show significant expansion until 2020. In comparison, camp 2 existed before 1995 and developed gradually after 2015. Camp 3 was newly identified in 2019 and expanded rapidly into the eastern and southern areas.

**Figure 6:**
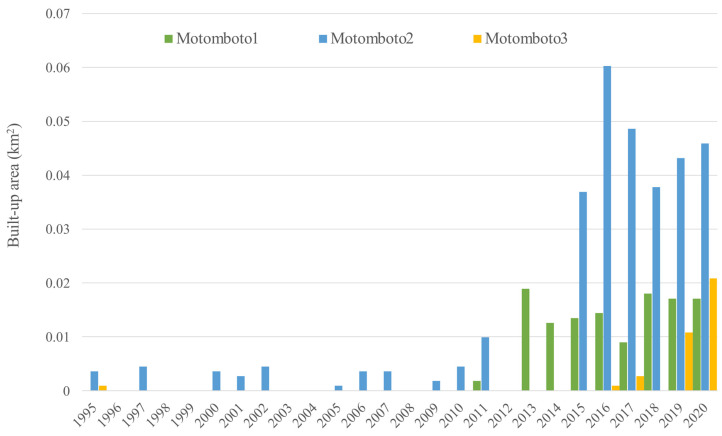
Built-up areas by ASGM camp.

From the results of NDVI, the built-up areas in the ASGM camps were extracted as described in Section 2.4. Together with various landcover changes, built-up areas were detected in all of the ASGM camps, with an accuracy of 96% (Figure 6). Built-up areas were identified from 2011, 1995, and 2016 in Motomboto ASGM camps 1, 2, and 3, respectively. The built-up area in camp 1 developed largely in 2013, but the area remained around the same size. Camp 2 mainly developed around 2015, and the largest area was detected in 2016. Camp 3 was clearly distinguishable from the others and showed a continuous rapid increase in size even after the Minamata Convention on Mercury was brought into force in 2017. Notably, in 2020, ASGM camp 3 showed a 23-fold increase in size over that in 2017. The built-up areas in the mining camps tended to be detected approximately 1 year after the landcover changes described in the previous paragraph were identified.

### 3.2. Investigation of ASGM Camps

**Figure 7:**
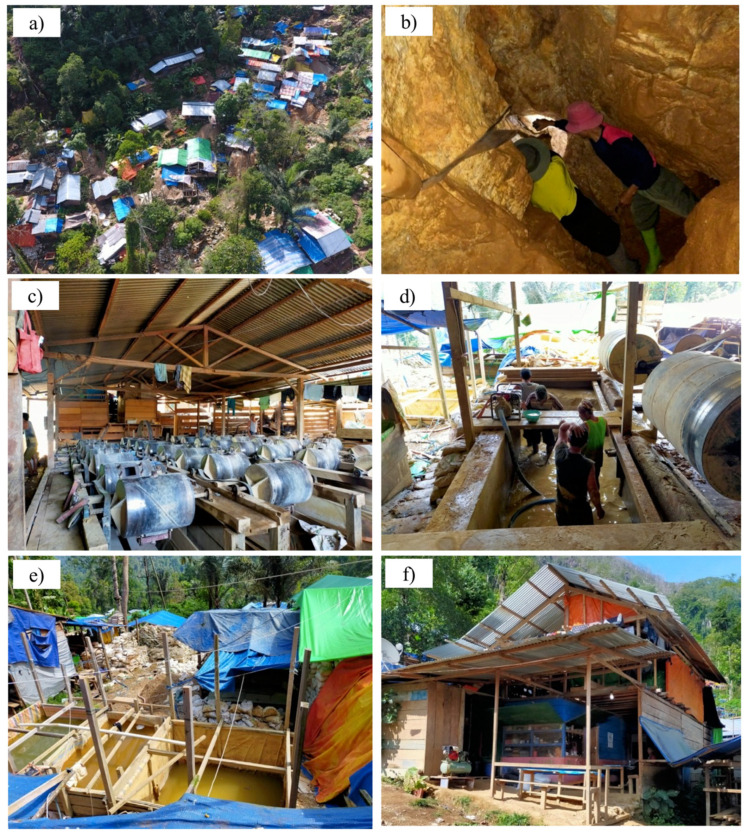
Motomboto ASGM camp. (**a**) Settlements in Motomboto ASGM 3 taken by an unmanned aerial vehicle. (**b**) An active mine hole. (**c**) Trommels for milling materials mined from holes. (**d**) A pool of water mixed with hydrogen peroxide for immersing the milled materials. (**e**) A pool of mercury mixed with cyanide for immersing the materials. (**f**) A common settlement where incoming miners and their families stay and manage a grocery shop.

Motomboto ASGM camp is located 4–6 h from the center of East Suwawa. There are no paved roads, and the camps are only accessible by motorcycle. The road conditions are poor, and miners are required to cross several rivers by motorcycles to reach the camps. The basic settlements in the camps are composed of tin roofs or are covered by tarpaulins and are spread over in the small valley, forming village-like settlements (Figure 7a). In these simple settlements, all the processes required for the gold extraction are performed, and the incoming miners also stay in these mining camps.

Based on our field investigations, the total number of miners in the Suwawa area was approximately 10,000. Because the miners tended to be replaced frequently, they were difficult to count accurately; however, 75–85% of the incoming miners were from rural areas, where they engaged in agricultural or fishery industries. At the ASGM sites, more than two teams were organized per tunnel, working in 24 h shifts (as reported by a local miner).

The activities conducted in the Motomboto ASGM camps are shown in Figure 7b–e. Mercury and cyanide are required in the process of gold amalgamation in the tailings. Figure 7f shows the residences of the incoming miners and their families, where the miners’ families generally operate small restaurants or grocery stores to service the influx miners and their families’ daily needs. The ASGM activity in this area has increased rapidly since 2017, with influxes of miners after the gold price increased (as reported by a local miner).

### 3.3. Relationship between the Built-Up Areas and the Gold Price

**Figure 8:**
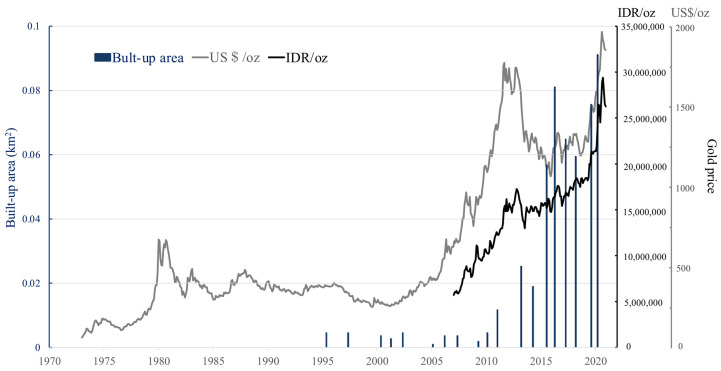
Global gold price, Indonesian gold price, and built-up areas in the camps.

The relationship between the built-up areas in the ASGM camps and the gold price was assessed (Figure 8). The built-up areas identified at the three mining sites were combined. The global gold price increased rapidly from 2006 to 2012. It then decreased and remained steady till early 2019, when it increased again until 2021. The gold price in Indonesia has increased since 2007, with an especially steadily increase since 2017, approximately doubling by late 2020. Although there are differences in the Indonesian and global gold prices, similar tends were observed. From 2007 to 2020, the increase in the built-up areas was significantly associated with the gold price (R^2^ = 0.91).

## 4. Discussion and Limitations

### 4.1. Discussion

The built-up areas developed in ASGM camps are indicators of transformations at the ASGM sites and, in this study, they provided a developmental time series that correlated with the changes in gold prices. A quantitative analysis of the ASGM sector extends our understanding of the rate of development of mining sites and their transformation over time. Understanding the status of ASGM is essential to tracking its responses to global factors, such as the gold price and the Minamata Convention on Mercury (2017), and to predicting the rate and level of socio-environmental destruction at the local and community levels.

Identification of proposed built-up areas, as an indicator of the growth of the camp-type ASGM, using Landsat series with 30 m ground resolution, demonstrated the transformation of camp-type ASGM over decades (Figure 3, Figure 4, Figure 5 and Figure 6), as reported previously [28]. With a quantitative time-series analysis, we detected various forms of built-up areas in the mining camps, indicative of the camps’ characteristics. For example, Motomboto camp 1 was identified as a newly opened area in 2011, whereas the mining activities at camp 2 expanded rapidly in 2015. Although, the development of camp 3 is considered recent, it developed rapidly after 2016. This expansion and development resulted from new influxes of miners and the weak enforcement of their regulation. Illegal immigrant miners from neighboring regions, including Bolaang Mongondow and Minahasa (North Sulawesi), have been reported [38]. The population of East Suwawa, where the targeted mining camps are located, did not show a significant change with the population in 2007–2019 [58,59,60,61,62,63,64,65,66,67,68]. Because local residents travel from their own villages near the mining camp (field interview), the development of the built-up areas in a mining camp reflects the increased influx of miners into that mining camp. Possible factors encouraging their entry include the weak regulation of the ASGM sector resulting from its informal, illegal, and closed nature [3]; limited governments resources and administrative capacity to provide adequate technical assistance or to enforce compliance [69]; and the remote locations of the mining sites [35]. The identification of policy problems and the development of planning and management solutions in remote rural locations further impede the proper management of the sector [70]. Because the Motomboto ASGM camps are located in remote rural areas in a national park, the regulation and monitoring of miner influxes from other regions and mining activities are more complex than those at easily accessible sites.

Our finding of a significant relationship between the developmental scale represented by built-up areas and the gold price in Indonesian rupiah is consistent with those previous studies [20,21]. Although mining types differ, similar trends have been detected between these two factors.

The living conditions in the mining camps can have negative social and health effects, resulting from the population’s exposure to high levels of mercury vapor. The emission of mercury from the camps into the atmosphere and its releases into Bone River, the main water supply for the city of Gorontalo, further harm both human and environment health at the community and regional levels. Other problems are expected inside the mining camps, such as a high incidence of infection among children, an increased prevalence of tropical diseases such as Dengue fever, and a lack of access to health care, education for children, safe water, wastewater treatment, and sanitation supplies, as reported in previous studies [71,72,73,74]. However, artificial camp-type AGSM sites, including the Motomboto sites do not generally contain any basic infrastructures, resulting in rapid environmental pollution owing to poor waste treatment.

### 4.2. Limitations

This study had several limitations associated with the quality of the input data. First, cloud-free Landsat series and complete images of Landsat7 were limited due to various factors, including scanline errors. Second, differences in the spatial resolution of the datasets used resulted in mixed pixels, possibly causing the overestimation or miscalculation of built-up areas. Third, the methodology used in this study is only applicable to similar camp-type mining sectors.

## 5. Conclusions

In this study, the transformation of the ASGM sector, with large influxes of miners living at mining camps in Bone Bolango Regency, Indonesia, was assessed with remote sensing imagery and field investigations. The results presented here show that the total built-up area in the target ASGM sites identified by Landsat series in 2020 had increased 18.6-fold relative to that in 1995 and correlated with the increase in the gold price in Indonesian rupiah. Furthermore, the large influx of miners living in mining camps paralleled the increase in the market price of gold. Therefore, we conclude that the spread of camp-type ASGM across a small remote area with large influxes of miners is detectable by monitoring the built-up areas in those mining camps. These results extend our understanding of the rate and scale of the development of the closed ASGM sector and provide significant insight into the potential for environmental pollution at the local and community levels. This will allow precautions to be taken and alternative sustainable strategies to be developed at the local, community, and regional levels.

## Figures and Tables

**Table 1:** Main specification of satellite imagery in the study.

Acquisition Date	Sensor	NIR (μm)	Red (μm)	Green (μm)
30 May 1995 26 May 1997 11 May 2000 4 April 2001 25 May 2002	Landsat 5 TM	0.70–0.80	0.60–0.70	0.50–0.60
27 March 2004 10 February 2005 17 March 2006, 25 September 2006 23 May 2007, 30 October 2007 26 April 2009 24 February 2010, 4 September 2010 10 January 2011, 26 November 2011 27 March 2013 24 April 2014	Landsat 7 ETM+	0.76–0.90	0.63–0.69	0.52–0.60
8 July 2015 5 April 2016 24 April 2017 10 March 2018 4 August 2019 15 March 2020	Landsat 8 OLI	0.85–0.88	0.64–0.67	0.53–0.59
